# Enhanced anti-tumour activity of the combination of the novel MEK inhibitor WX-554 and the novel PI3K inhibitor WX-037

**DOI:** 10.1007/s00280-016-3186-4

**Published:** 2016-11-11

**Authors:** Emma J. Haagensen, Huw D. Thomas, Wolfgang A. Schmalix, Andrew C. Payne, Lara Kevorkian, Rodger A. Allen, Paul Bevan, Ross J. Maxwell, David R. Newell

**Affiliations:** 1 Newcastle Cancer Centre, Northern Institute for Cancer Research, Paul O’Gorman Building, Medical School, Newcastle University, Framlington Place, Newcastle-upon-Tyne, NE2 4HH UK; 2 Wilex AG, Grillparzerstrasse 18, 81675 Munich, Germany; 3 UCB Pharma Ltd, 208 Bath Road, Slough, SL1 3WE UK

**Keywords:** PI3K, MEK, Combination, Synergy; Colorectal cancer

## Abstract

**Purpose:**

Tumours frequently have defects in multiple oncogenic pathways, e.g. MAPK and PI3K signalling pathways, and combinations of targeted therapies may be required for optimal activity. This study evaluated the novel MEK inhibitor WX-554 and the novel PI3K inhibitor WX-037, as single agents and in combination, in colorectal carcinoma cell lines and tumour xenograft-bearing mice.

**Methods:**

In vitro growth inhibition, survival and signal transduction were measured using the Sulforhodamine B, clonogenic and Western blotting assays, respectively, in HCT116 and HT29 cell lines. In vivo anti-tumour efficacy and pharmacokinetic properties were assessed in HCT116 and HT29 human colorectal cancer xenograft tumour-bearing mice.

**Results:**

The combination of WX-554 and WX-037 exhibited marked synergistic growth inhibition in vitro, which was associated with increased cytotoxicity and enhanced inhibition of ERK and S6 phosphorylation, compared to either agent alone. Pharmacokinetic analyses indicated that there was no PK interaction between the two drugs at low doses, but that at higher doses, WX-037 may delay the tumour uptake of WX-554. In vivo efficacy studies revealed that the combination of WX-037 and WX-554 was non-toxic and exhibited marked tumour growth inhibition greater than observed with either agent alone.

**Conclusion:**

These studies show for the first time that combination treatment with the novel MEK inhibitor WX-554 and the novel PI3K inhibitor WX-037 can induce synergistic growth inhibition in vitro, which translates into enhanced anti-tumour efficacy in vivo.

**Electronic supplementary material:**

The online version of this article (doi:10.1007/s00280-016-3186-4) contains supplementary material, which is available to authorized users.

## Introduction

The development of numerous targeted small molecule inhibitors represents an important and evolving new approach to cancer therapy. However, as tumours often have defects in multiple oncogenic signalling pathways, single agent anti-tumour activity is modest, and thus combinations of targeted agents are being investigated. Specifically, the MAPK pathway, a major proliferative pathway, and the PI3K pathway, a major survival pathway, are frequently activated in cancer and are being concomitantly targeted.

Many MEK inhibitors, such as PD 0325901 and selumetinib (AZD6244), have been developed to target the MAPK pathway and have shown potent growth inhibitory activity in experimental systems [1–4]. A novel orally available small molecule allosteric MEK inhibitor WX-554 (UCB1366554), which potently inhibits MEK1 and MEK2 with a half maximal inhibitory concentration (IC_50_) of 4.7 and 11 nM, respectively, has been developed by Wilex and UCB Celltech. WX-554 has demonstrated marked inhibition of ERK1/2 phosphorylation in HT29 cells, and growth inhibition in a range of cell lines in vitro, and tumour growth delay or stasis in vivo, with increased sensitivity in BRAF or RAS mutant cells and tumours [5]. WX-554 has been shown to be safe and tolerable in a dose escalation study in healthy volunteers [6], and a recent Phase I study indicated that WX-554 has very good bioavailability and that it is able to inhibit MEK signal transduction in a dose-dependent manner [3, 7, 8]. WX-554 was investigated in a Phase Ib/II dose escalation study to determine safety and pharmacokinetics/pharmacodynamics in patients with solid tumours [7, 9], which was terminated due to business, rather than clinical, reasons.

Targeting the PI3K/AKT pathway has been investigated with a range of PI3K inhibitors which can be isoform-specific inhibitors, pan class I inhibitors (e.g. pictilisib, GDC-0941) or dual PI3K/mTOR inhibitors (e.g. dactolisib, NVP-BEZ235), and promising anti-cancer efficacy has been reported in pre-clinical models [10–16]. Wilex and UCB Celltech have also developed a novel small molecule pan class I PI3K inhibitor, WX-037 (UCB1370037), from an indole series. WX-037 is a potent inhibitor of the α and δ isoforms of PI3K (IC_50_ = 4.1 and 2.4 nM, respectively) and a weaker inhibitor of the β and γ isoforms of PI3K and DNA-PK (IC_50_ = 69, 36 and 28 nM, respectively) with no detectable inhibition of mTOR (IC_50_ = >20,000 nM). In pre-clinical studies, WX-037 demonstrated strong inhibition of AKT phosphorylation, and promising growth inhibition in a range of cell lines in vitro, and tumour growth delay or stasis in vivo, with greater sensitivity observed in PIK3CA mutant or PTEN null cells and tumours [5]. WX-037 was investigated in a Phase I dose escalation study to investigate its safety, pharmacokinetics, pharmacodynamics and clinical activity in patients with solid tumours; however, this trial was also terminated due to business reasons [9, 17].

Previous studies have shown that concomitant inhibition of the PI3K and MAPK pathways by PI3K and MEK inhibition yields promising anti-cancer effects in vitro and in vivo [2, 18–26], and the combination of WX-554 and WX-037 demonstrated synergy in vitro and increased efficacy in vivo [5]. Consequently, the Phase I trial of WX-037 was designed not just to investigate the efficacy of single agent WX-037, but also the combination of WX-554 and WX-037 [9].

The aim of this study was to determine the in vitro and in vivo activity of the novel MEK inhibitor WX-554 and the novel PI3K inhibitor WX-037, alone and in combination, in colorectal carcinoma cell lines and tumour xenograft-bearing mice. These colorectal carcinoma cell lines (HT29 and HCT116) were used as they both contain KRAS/RAF and PI3K pathway mutations. The first objective was to determine the in vitro potency and efficacy of the compounds, alone and in combination, by measuring growth inhibition using an SRB assay, cytotoxicity via a clonogenic assay and cell signalling by Western blotting, and to investigate if the effects of the combinations on cell growth were synergistic, additive or antagonistic using median effect analyses. These results were then used to design in vivo experiments to investigate the pharmacokinetic profile of the compounds and their efficacy, alone and in combination.

## Methods

### Ethics statement

All in vivo experiments were reviewed and approved by the Newcastle University (UK) animal welfare committee and were performed according to the guidelines for the welfare and use of animals in cancer research [27] and national law, under project license (PPL60/4442) issued by the UK Government Home Office under the animals (scientific procedure) act 1986.

### Inhibitors

The MEK inhibitor WX-554 and the PI3K inhibitor WX-037 were kindly supplied by Wilex, Munich, Germany. For in vitro studies, the inhibitors were dissolved in anhydrous dimethyl sulphoxide (DMSO) and were stored frozen under light-protected conditions at −20 °C. For in vivo studies, the MEK inhibitor WX-554 was dissolved in 0.9% NaCl (w/v), 10 mM Na-citrate pH 3.0 (w/v) and 0.2% Tween 20 (v/v) in sterile distilled water and the PI3K inhibitor WX-037 was suspended in SMEDDS (self-microemulsifying drug delivery system; 25% Capmul MCM EP (glycerol monocaprylocaprate) (v/v), 37.5% Tween 80 (polyoxyethylene(20) sorbitan monooleate) (v/v) and 37.5% PEG 400 (polyethylene glycol 400) (v/v)).

### Cell lines and reagents

HCT116 and HT29 human colorectal cancer cells were obtained from the ATCC (American Type Culture Collection). All cell lines were grown in RPMI-1640 medium (supplemented with 10% (v/v) foetal bovine serum, 1% (v/v) penicillin (50 U/ml)—streptomycin (50 mg/ml) and 2 mM L-glutamine) and were confirmed free of mycoplasma contamination by regular testing with Mycoalert (Cambrex, Iowa, USA).

### Animals

Animal studies were all carried out using female athymic CD1 nude mice (Charles River, Kent, UK), implanted with HCT116 or HT29 xenografts (1 × 10^7^ cells in 50 µl media injected subcutaneously into the right flank), maintained and handled in isolators under specific pathogen-free conditions.

### Growth inhibition assay

Exponentially growing cells were grown in media in 96-well format and were exposed to increasing concentrations of the single agent inhibitors WX-554 or WX-037, or WX-554 combined with WX-037 at 0.25, 0.5, 1, 2 or 4 times their half maximal growth inhibitory concentration (GI_50_) in DMSO, or 0.5% DMSO alone, for 72 h. Growth was then measured using the Sulforhodamine B (SRB) method and analysed as described previously [23]. The GI_50_ concentration was calculated based on a standard point to point curve with 1000 segments using GraphPad Prism software (California, USA). The data were analysed by median effect analysis using CalcuSyn software (Biosoft, Cambridgeshire, UK), which calculates the combination index of multiple drugs by an algebraic estimation algorithm.

### Cytotoxicity assay

Exponentially growing cells were exposed to increasing concentrations of the single agent inhibitors WX-554 or WX-037, or 10 µM of WX-554 combined with 10 µM WX-037 in DMSO or 0.5% (v/v) DMSO alone for 72 h before harvesting and reseeding for colony formation. After growth for 10–14 days, colonies were fixed in methanol–acetic acid 3:1 (v/v) and stained with crystal violet (0.4% w/v). Colonies consisting of more than 50 cells were counted on an automated colony counter (Oxford Optronix, Oxford, UK). Two-tailed paired t tests were used to compare the different groups. Differences with a *p* < 0.05 were considered statistically significant.

### Western blotting

Cells were treated with WX-554 and WX-037 at 1 or 10 times the half maximal growth inhibitory concentration (GI_50_) in DMSO, or 0.5% (v/v) DMSO alone, for 24 h. Western blots were prepared, probed with phospho-4EBP1 (Thr37/46) (#2855), phospho-p44/42 MAPK (Thr202/Tyr204) (#4370), phospho-AKT (Ser473) (#4060) or phospho-S6 ribosomal protein (Ser235/236) (#4858) monoclonal antibodies obtained from Cell Signalling Technology (New England BioLabs (UK) Ltd, Hertfordshire, UK) and developed as described previously [23]. Blots were then stripped (100 mM 2-mercaptoethanol, 2% (w/v) SDS and 62.5 mM Tris pH6.8 at 55 °C for 30 min) and re-probed with the respective total monoclonal antibody (4EBP1 (53H11) (#9644), p44/42 MAPK (ERK1/2) (#4695), AKT (pan) (C67E7) (#4691) or S6 ribosomal protein (5g10) (#2217)) obtained from Cell Signalling Technology (New England BioLabs (UK) Ltd, Hertfordshire, UK) and developed as described above.

### Pharmacokinetic (PK) studies

Mice bearing HCT116 or HT29 human tumour xenografts were treated with 1 or 5 mg/kg WX-554, or 20 or 100 mg/kg WX-037, alone or in combination, and were bled by cardiac puncture under terminal anaesthesia at 6 or 24 h post-treatment (3 mice/time point). Blood was collected into heparinized tubes, and plasma was separated and stored at −20 °C until analysed. Tumours were removed, snap frozen in liquid nitrogen and stored at −80 °C prior to PK analyses. Samples were extracted with solid phase extraction (SPE) and analysed with high-performance liquid chromatography (HPLC) coupled with tandem mass spectrometry (MS/MS) by Wilex (Munich, Germany). The quantification limit for WX-554 was 1 ng/mL and within and between day variation was <15%. WinNonlin Software Version 4.0.1 (Pharsight Corporation, Peypin, France) was used for PK/PD modelling and non-compartmental analysis. Paired t tests were used to compare the different treatment groups, and differences with a *p* value ≤0.05 were considered statistically significant.

### Determination of anti-tumour activity

Mice bearing HCT116 human tumour xenografts were randomized into treatment groups and then treated by oral gavage with either the vehicle (10 ml/kg), 2 mg/kg WX-554, 50 mg/kg WX-037 or the combination of 2 mg/kg WX-554 and 50 mg/kg WX-037 once daily for 14 days. Tumour volume was monitored by calliper measurement using the equation *a* ^2^ × *b*/2, where *a* is the smallest measurement and *b* the largest. Data are presented as median relative tumour volumes (RTV), where the tumour volume in each mouse on the initial day of treatment (day 0) is assigned an RTV value of 1. The time to RTV4 for each individual tumour was calculated based on a standard point to point curve with 1000 segments using GraphPad Prism software (CA, USA). Mann–Whitney U tests were used to compare the different groups, i.e., the control versus each treatment group, the single agents versus each other and each single agent versus their combination. Differences with a *p* value ≤0.05 were considered statistically significant.

## Results

### The PI3K inhibitor WX-037 and the MEK inhibitor WX-554 are synergistic and exhibit increased cytotoxicity in combination in vitro

The growth inhibitory activity of the PI3K inhibitor WX-037 and the MEK inhibitor WX-554, as single agents, in HCT116 and HT29 cells was measured using the SRB assay (Supplementary Figure 1). Both drugs induced over 65% growth inhibition in both the colorectal cell lines. The results were used to determine the half maximal growth inhibitory (GI_50_) concentration of the drugs after 72-h exposure. The MEK inhibitor WX-554 was found to have GI_50_ values of 38 and 4.3 nM, whereas the PI3K inhibitor WX-037 was less potent with GI_50_ values of 2934 and 112 nM in the HCT116 and HT29 cell lines, respectively (Supplementary Figure 1).

**Fig. 1:**
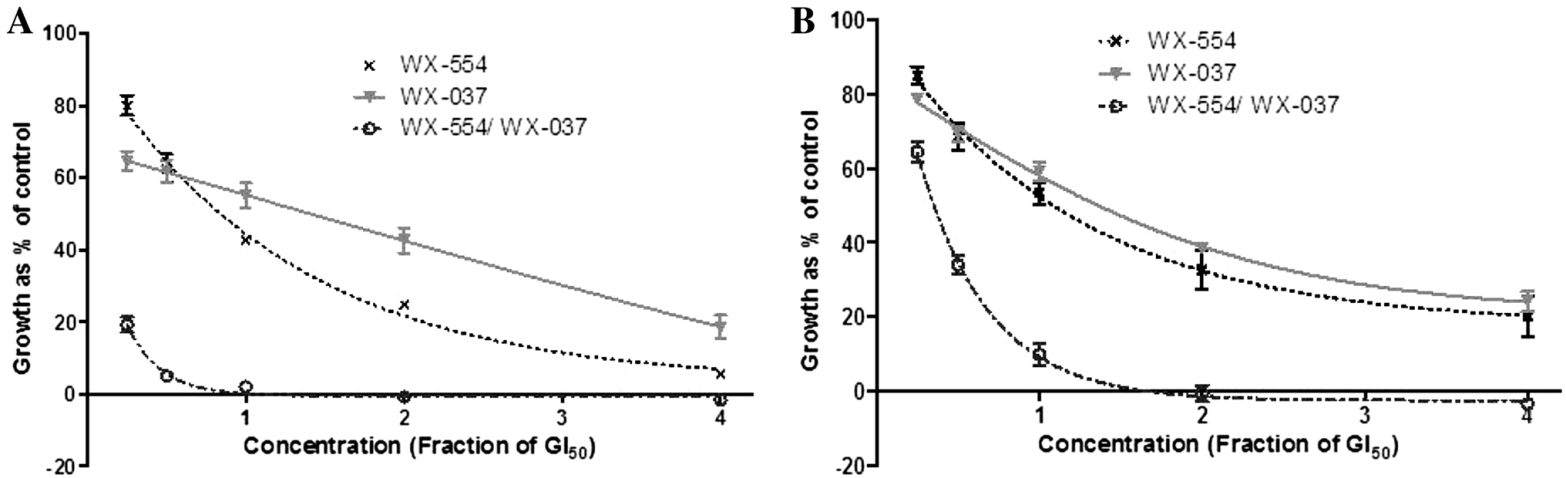
Growth inhibition induced by the PI3K inhibitor WX-037 and the MEK inhibitor WX-554, alone and in combination, in the HCT116 and HT29 cell lines. HCT116 (**a**) and HT29 (**b**) cells were treated with the indicated fractions of the GI_50_ concentrations of the inhibitors, alone or in combination, derived from Supplementary Figure 1, for 72 h, and an SRB assay was subsequently performed. Growth is presented as a percentage of the control, in which cells were treated with 0.5% (v/v) DMSO. Points represent the mean of 3 independent experiments ± standard error. *Lines* were fitted using nonlinear regression analysis

Studies were then performed to determine the effect of combining the PI3K and MEK inhibitors on colorectal carcinoma cell growth over 72 h. WX-037 and WX-554 were used alone at 0.25x, 0.5x, 1x, 2x and 4x their respective GI_50_ concentration, as calculated from Supplementary Figure 1, and at equipotent concentrations at the same GI_50_ ratios in combination. Figure 1 shows that the combination of WX-037 and WX-554 was markedly more growth inhibitory than either compound alone, completely inhibiting growth at the highest concentrations. Data were then evaluated by median effect analysis (CalcuSyn, Biosoft, Great Shelford, UK) to determine whether the greater growth inhibitory activity of the combination of WX-554 and WX-037 reflected an additive or a synergistic effect. The combination of the PI3K inhibitor WX-037 and the MEK inhibitor WX-554 was strongly synergistic when combined at the GI_50_ concentration compared to the compounds alone (Supplementary Table 1).

Cell survival after 72-h exposure to the PI3K inhibitor WX-037 and the MEK inhibitor WX-554 was also measured using a clonogenic cytotoxicity assay. Single agent WX-554 showed significant cytotoxicity at 10 µM with 67% cell kill in the HCT116 cell line and 75% in the HT29 cell line; however, the mean lethal concentration (LC_50_) of 0.6 µM and 1.6 µM WX-554 was approximately 16-fold and 372-fold higher than the corresponding GI_50-_ values in the HCT116 and HT29 cell lines, respectively. WX-037 showed no marked cytotoxicity with less than 50% cell death after 72 h treatment at 10 µM (Supplementary Figure 2).

**Fig. 2:**
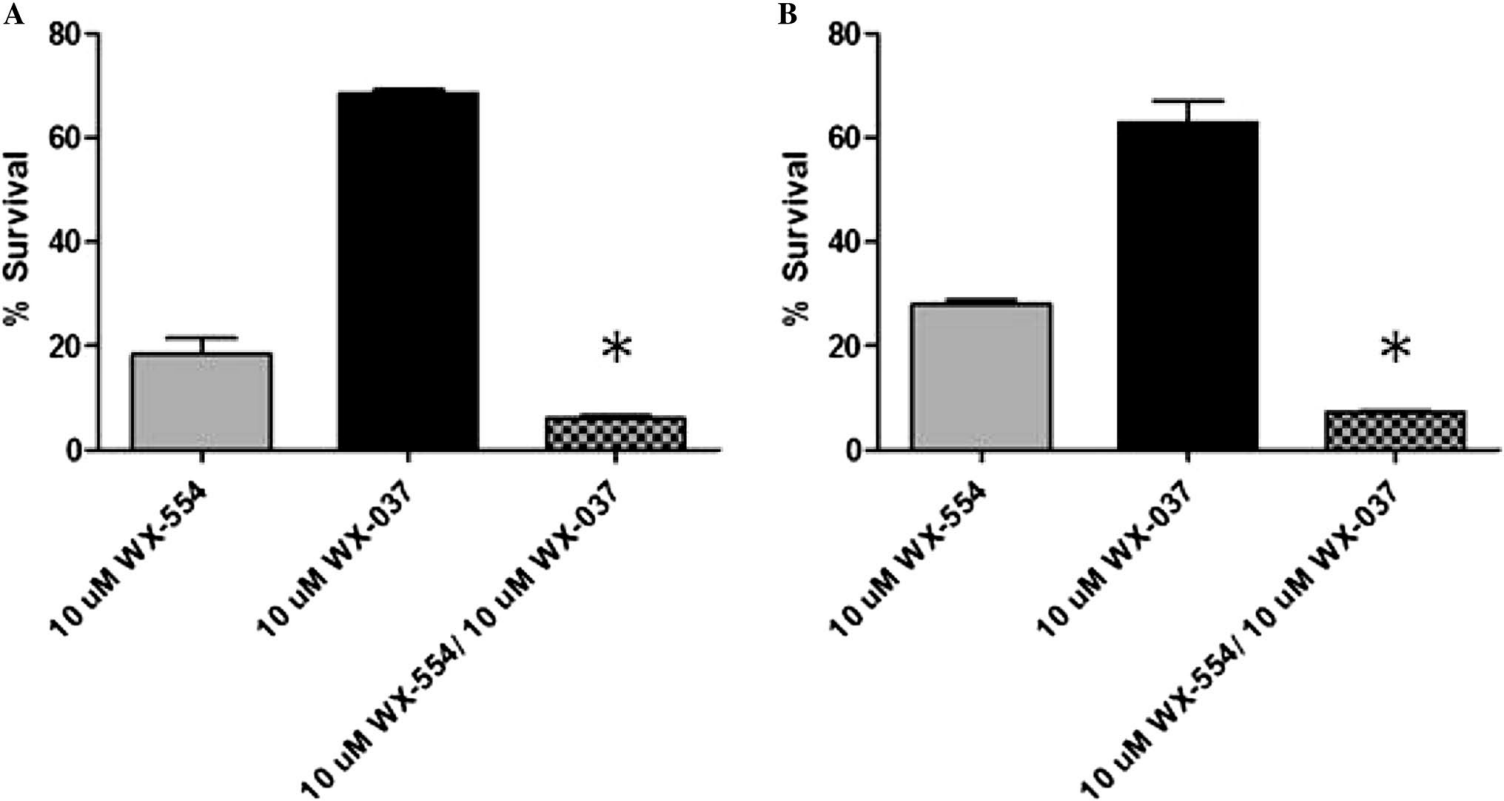
Cell survival after 72-h exposure to 10 µM of the PI3K inhibitor WX-037 and 10 µM of the MEK inhibitor WX-554, alone and in combination, in the HCT116 cell line and HT29 cell lines. HCT116 (**a**) and HT29 (**b**) cells were treated with a fixed concentration of each inhibitor alone or in combination for 72 h, and cell survival was subsequently determined by clonogenic assay after 10–14 days of colony growth. Survival is presented as a percentage of the control, in which cells were treated with 0.5% (v/v) DMSO. *Bars* represent the mean of 3 independent replicates ± standard error. *Significantly different from either agent alone, *p* ≤ 0.05

The cytotoxicity of the PI3K and MEK inhibitors in combination after 72 h treatment was then determined. However, as WX-037 did not produce > 50% cytotoxicity at 10 µM, it was not possible to determine an LC_50_ value, and hence the highest concentration previously used of 10 µM WX-037 was combined with 10 µM WX-554. There was a statistically significant increase in cytotoxicity when the PI3K and MEK inhibitors were combined, compared to the cytotoxicity induced by the drugs as single agents, in the HCT116 (*p* = 0.02) and HT29 (*p* < 0.01) cell lines (Fig. 2). Overall, the interaction of WX-037 and WX-554 resulted in significantly enhanced cell growth inhibition and an increase in cytotoxicity in both cell lines studied.

The effect of 24-h exposure to the PI3K inhibitor WX-037 and the MEK inhibitor WX-554, both as single agents and in combination, was also investigated by Western blotting to determine the effect on the PI3K/AKT signalling pathway, using total and phospho-specific antibodies for AKT, S6 and 4EBP1 and the effect on MAPK signalling, using total and phospho-specific antibodies for ERK1/2. The compounds were used as single agents or in combination at their respective GI_50_ concentrations and at 10x the GI_50_ concentration.

Supplementary Figure 3 shows that treatment with the MEK inhibitor WX-554 reduced ERK1/2 phosphorylation at 1 and 10 times the GI_50_ concentration in the HCT116 cell line, and at 10 times the GI_50_ concentration in the HT29 cell line. The reduction in ERK1/2 phosphorylation was enhanced with the combination of WX-554 and WX-037 leading to complete inhibition with 10 times the GI_50_ concentration in both colorectal carcinoma cell lines (Supplementary Figure 3). Additionally, there was a concentration-dependent reduction in AKT phosphorylation in the HCT116 cell line after treatment with the single agent PI3K inhibitor WX-037 which was enhanced, to yield complete inhibition at the GI_50_ concentration, after combined WX-037 and WX-554 treatment. In the HT29 cell line, there was a reduction in AKT phosphorylation at 10 times the GI_50_ concentration; however, this reduction was similar with single agent WX-037 and the combination (Supplementary Figure 3).

Single agent WX-037 also caused a concentration-dependent reduction in S6 phosphorylation at 1 and 10 times the GI_50_ concentration, and treatment with the combination of WX-037 and WX-554 enhanced this inhibition, causing complete inhibition at 10 times the GI_50_ concentration in both colorectal carcinoma cell lines (Supplementary Figure 3). However, WX-037 had no marked effect on the phosphorylation of 4EBP1 alone or in combination with WX-554 in either colorectal carcinoma cell line (Supplementary Figure 3). Hence, overall, the combination of the MEK inhibitor WX-554 and the PI3K inhibitor WX-037 resulted in enhanced inhibition of ERK1/2 and S6 phosphorylation, and inhibition of AKT phosphorylation, in both colorectal carcinoma cell lines. However, both the single agents and the combination had no major impact on 4EBP1 phosphorylation.

### The PI3K inhibitor WX-037 and the MEK inhibitor WX-554 exhibit increased tumour growth delay in combination in vivo

**Fig. 3:**
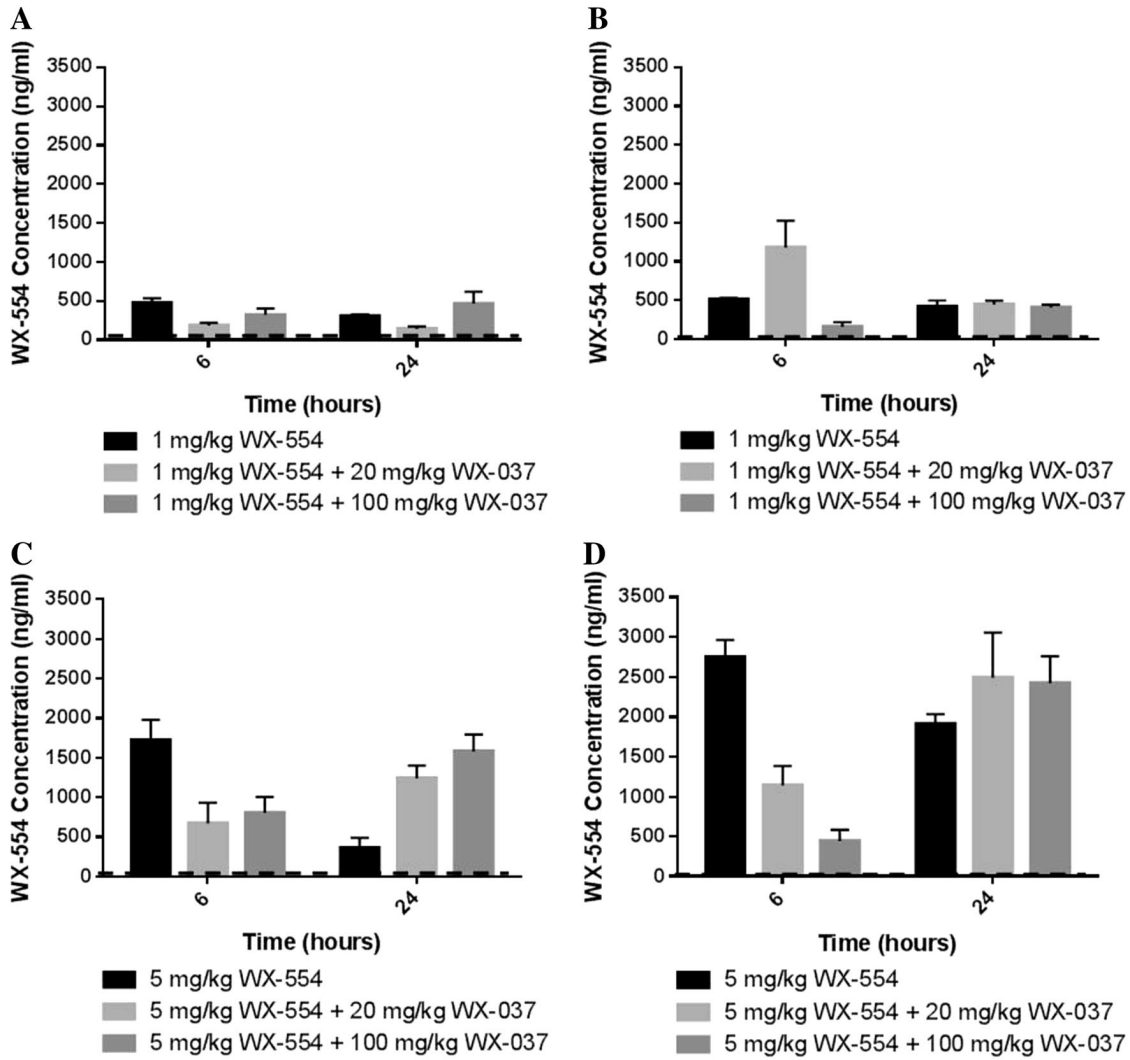
Concentrations of the MEK inhibitor WX-554 alone and in combination with the PI3K inhibitor WX-037 in tumours from mice bearing HCT116 or HT29 human tumour xenografts. Tumour concentrations of WX-554 measured by LC–MS/MS from HCT116 (**a**, **c**) and HT29 (**b**, **d**) tumour xenograft-bearing mice at the indicated time points after a single p.o. dose of 1 mg/kg (**a**, **b**) or 5 mg/kg (**c**, **d**) WX-554 alone or combined with 20 or 100 mg/kg WX-037. Data are presented as the mean concentration from 3 mice in each group ± standard error. *Horizontal dashed lines* indicate the in vitro GI_50_ concentration for the respective cell line, calculated from Supplementary Figure 1

**Fig. 4:**
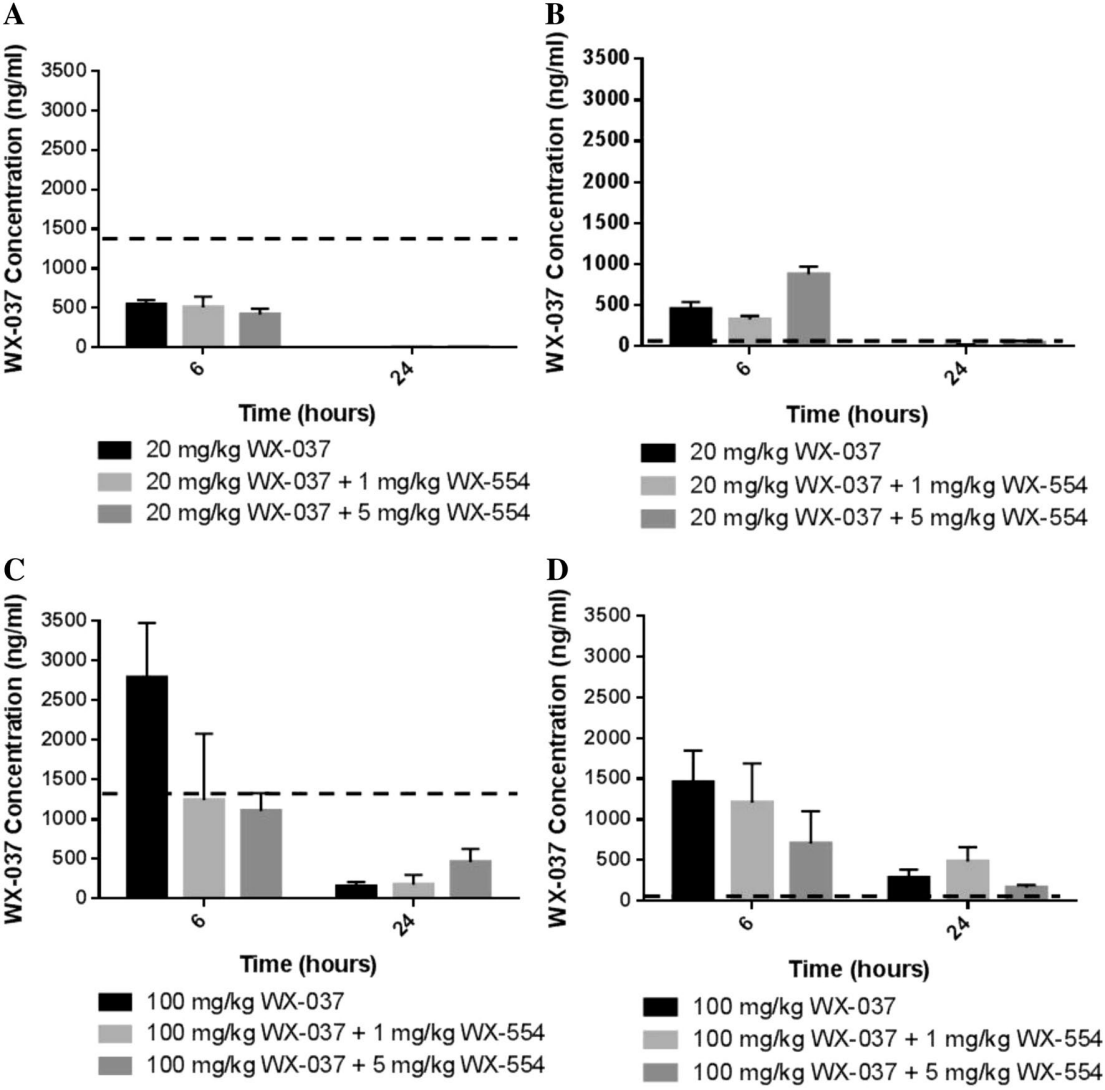
Concentrations of the PI3K inhibitor WX-037 alone and in combination with the MEK inhibitor WX-554 in tumours from mice bearing HCT116 or HT29 human tumour xenografts. Tumour concentrations of WX-037 measured by LC–MS/MS from HCT116 (**a**, **c**) and HT29 (**b**, **d**) tumour xenograft-bearing mice at the indicated time points after a single p.o. dose of 20 mg/kg (**a**, **b**) or 100 mg/kg (**c**, **d**) WX-037 alone or combined with 1 or 5 mg/kg WX-554. Data are presented as the mean concentration from 3 mice in each group ± standard error. *Horizontal dashed lines* indicate the in vitro GI_50_ concentration for the respective cell line, calculated from Supplementary Figure 1

A PK study was carried out with samples taken 6 and 24 h after treatment with a single dose of the PI3K inhibitor WX-037 and the MEK inhibitor WX-554, alone and in combination, in HCT116 and HT29 human tumour xenograft-bearing mice. The concentrations of the drugs in the plasma and the tumour tissue were measured using LC–MS/MS (Figs. 3, 4, Supplementary Figures 4 and 5 and Supplementary Table 2).

After a single dose of 1 or 5 mg/kg WX-554, alone or in combination with 20 or 100 mg/kg WX-037, WX-554 concentrations in the plasma and tumour tissue generally greatly exceeded the in vitro GI_50_ value of 18 ng/ml (38 nM) in the HCT116 cell line and 2 ng/ml (4 nM) in the HT29 cell line (determined in Supplementary Figure 1) (Fig. 3, Supplementary Figure 4 and Supplementary Table 2A). The exception to this was that plasma WX-554 concentrations were only approximately equal to the HCT116 GI_50_ value at 6 h and were below the HCT116 GI_50_ value by 24 h, in the HCT116 tumour xenograft-bearing mice after treatment with 1 mg/kg WX-554, alone or in combination with 20 or 100 mg/kg WX-037 (Supplementary Figure 4A and Supplementary Table 2A). Additionally, the plasma WX-554 concentration was also below the HCT116 GI_50_ value by 24 h after 5 mg/kg WX-554 alone; however, WX-554 concentrations still exceeded the GI_50_ value when 5 mg/kg WX-554 was combined with WX-037 (Supplementary Figure 4C and Supplementary Table 2A). However, overall, the absolute plasma and tumour levels of WX-554 were generally similar in the HT29 and HCT116 tumour xenograft-bearing mice (Fig. 3, Supplementary Figure 4 and Supplementary Table 2A).

There was no consistent effect of concomitant dosing with 20 or 100 mg/kg WX-037 on the levels of WX-554 in the tumour or the plasma after administration of 1 mg/kg WX-554 in either HCT116 or HT29 tumour xenograft-bearing mice. In contrast, after dosing with 5 mg/kg WX-554, there was generally a WX-037 dose-dependent decrease in the levels of WX-554 at 6 h in the tumour and the plasma upon concomitant treatment with WX-037, which was significant for the tumour data at 6 h (*p* < 0.05) (Fig. 3, Supplementary Figure 4 and Supplementary Table 2A). These results suggest that WX-037 may delay the tumour uptake of WX-554 at the higher dose (5 mg/kg) compared to when WX-554 is administered alone.

In contrast to the data for WX-554, after treatment with 20 mg/kg WX-037, as a single agent or in combination with 1 or 5 mg/kg WX-554, WX-037 concentrations in plasma and tumour tissue at 6 and 24 h were markedly lower than the in vitro GI_50_ value of 1414 ng/ml (2934 nM) in the HCT116 cell line (determined in Supplementary Figure 1) (Fig. 4a, Supplementary Figure 5A and Supplementary Table 2B). Plasma and tumour WX-037 concentrations were also markedly below the HCT116 GI_50_ value 24 h after treatment with 100 mg/kg WX-037, given as a single agent or in combination with 1 or 5 mg/kg WX-554; however, concentrations in the plasma and tumour tissue were similar to or exceeded the HCT116 GI_50_ value 6 h after a 100 mg/kg dose of WX-037 (Fig. 4c, Supplementary Figure 5C and Supplementary Table 2B).

After treatment with 20 or 100 mg/kg WX-037, as a single agent or in combination with 1 or 5 mg/kg WX-554, concentrations in plasma and tumour tissue greatly exceeded the in vitro GI_50_ value of 54 ng/ml (112 nM) in the HT29 cell line (determined in Supplementary Figure 1) at 6 h. At 24 h, although concentrations were similar to or exceeded the HT29 GI_50_ value in all tumour samples and in plasma after a 100 mg/kg dose of WX-037, plasma concentrations had generally declined to below the GI_50_ value after a 20 mg/kg dose of WX-037 (Fig. 4b–d, Supplementary Figure 5B and D and Supplementary Table 2B). As with the WX-554 data, the absolute plasma and tumour levels of WX-037 were similar in the HCT116 and HT29 tumour xenograft-bearing mice (Fig. 4, Supplementary Figure 5 and Supplementary Table 2B).

There did not appear to be a consistent effect of concomitant dosing with 1 or 5 mg/kg WX-554 on the levels of WX-037 in the tumour or the plasma at 6 h after dosing with 20 mg/kg in either HCT116 or HT29 tumour xenograft-bearing mice (Fig. 4a, b, Supplementary Figure 5A and B and Supplementary Table 2B). However, after dosing with 100 mg/kg, there appeared to be a dose-dependent decrease in the levels of WX-037 in the tumour and the plasma upon concomitant dosing with WX-554 at 6 h, but this effect was only significant in HCT116 tumours (Fig. 4c, d, Supplementary Figure 5C and D and Supplementary Table 2B). Hence, concentrations of WX-037 were generally similar regardless of whether it was administered as a single agent or in combination with WX-554, and levels were consistently higher at 6 h compared with 24 h.

As the combination of WX-554 and WX-037 was synergistic in the in vitro studies, it may not be necessary for the drug concentrations in the plasma and the tumour to exceed those of the in vitro GI_50_ values for the single agents to achieve efficacy with the combination in vivo. Based on the in vitro results, in order to achieve half maximal growth inhibition, less than 1/6th and approximately 1/3rd of the single agent GI_50_ was required for 50% growth inhibition with the drug combination in the HCT116 and HT29 cell lines, respectively, which equates to GI_50_ values of 3 and <1 ng/ml WX-554 and 219 and 20 ng/ml WX-037 in the HCT116 and HT29 cell lines (calculated from Fig. 1). Therefore, with all the combinations of WX-554 and WX-037, WX-554 levels in the plasma and tumour tissues are at or exceed the GI_50_ concentration for the combination at both 6 and 24 h (Fig. 3, Supplementary Figures 4 and Supplementary Table 2A). Furthermore, with all combinations of WX-554 and 100 mg/kg WX-037, WX-037 levels in the plasma and tumour tissues are at or exceed the GI_50_ for the combination at both 6 and 24 h, and with all combinations of WX-554 with 20 mg/kg WX-037, WX-037 levels in the plasma and tumour tissues are at or exceed the GI_50_ for the combination at 6 h, but remain below at 24 h (Fig. 4, Supplementary Figure 5 and Supplementary Table 2B).

**Fig. 5:**
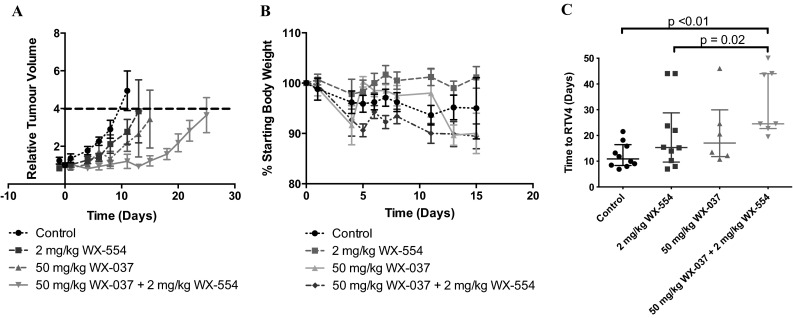
Efficacy and tolerability of the PI3K inhibitor WX-037 and the MEK inhibitor WX-554 in mice bearing human HCT116 colorectal tumour xenografts. HCT116 tumour xenografts were treated with either vehicle control, 2 mg/kg of the MEK inhibitor WX-554 and 50 mg/kg of the PI3K inhibitor WX-037 alone, or 2 mg/kg of the MEK inhibitor WX-554 and 50 mg/kg of the PI3K inhibitor WX-037 in combination, p.o. once daily for 14 days. **a** Tumour growth curves: data are presented as the median relative tumour volume (RTV), where the growth is calculated for each tumour relative to its size on day 0. Points represent the median of the 10 mice in each group. The *dashed line* shows the point at which tumours reached four times the initial volume (RTV4). **b** Effects on body weight: data are presented as a percentage of starting body weight. *Points* represent the mean of the mice in each group ± standard error. **c** Time taken for xenografts to reach four times the initial volume (time to RTV4): data are presented as the time taken by each individual tumour in each group to quadruple in size, and lines to represent the mean of the mice in each group ± standard error. *p* values are given where the combination is significantly different from either agent alone (*p* ≤ 0.05)

Based on the results of the PK study, the efficacy of 50 mg/kg of the PI3K inhibitor WX-037 and 2 mg/kg of the MEK inhibitor WX-554 given orally, as single agents and in combination, was assessed in HCT116 human tumour xenograft-bearing mice (Fig. 5). The individual doses of the PI3K and MEK inhibitors were chosen to be approximately equiactive, in order to mirror the in vitro conditions under which synergy had been demonstrated (Supplementary Figure 1). In this study, mice were treated daily for 14 days and tumour volumes were measured three times a week. Figure 5a demonstrates that treatment with 50 mg/kg WX-037 and 2 mg/kg WX-554, alone and in combination, caused tumour growth delay compared to vehicle-treated control tumours, and that growth delay was greater with the combination. Additionally, body weight was monitored daily to assess the tolerability of the therapy, and both single agent and combination treatments were found to be well tolerated as average body weights did not drop below 89% of the starting weight (Fig. 5b). The time for the tumours to quadruple in size (time to RTV4) was calculated (Fig. 5c), and statistical analyses using a Mann–Whitney test demonstrated a significant difference between vehicle-treated control tumours and the combination group (*p* < 0.01), and between the single agent MEK inhibitor and the combination group (*p* = 0.02).

## Discussion

The novel PI3K inhibitor WX-037 and the novel MEK inhibitor WX-554 have demonstrated in vitro activity in a range of cancer cell lines including breast, fibrosarcoma, thyroid, melanoma, colorectal, ovarian and pancreas lines with a broad range of GI_50_ values, where generally the cell lines most sensitive to WX-037 had PIK3CA mutations or PTEN loss, and to WX-554 had *BRAF* or *RAS* mutations [5]. Furthermore, the potency of WX-554 determined in the HCT116 and HT29 cell lines in this study (38 and 4.3 nM, respectively) was similar to that determined in the unpublished Wilex studies (29 and 7.2 nM) [5], and to the MEK inhibitor, PD 0325901 (21 and 6.5 nM) using the same assay [23]. Similarly the potency of WX-037 determined here in the HCT116 and HT29 cell lines (2934 and 112 nM, respectively) was similar to that reported in the unpublished Wilex studies (136 nM in the HT29 cell line) [5], and to the pan class I PI3K inhibitor, pictilisib (1081 and 157 nM) using the same assay [23].

In these in vitro studies, the HT29 colorectal carcinoma cell line was consistently more sensitive to WX-037 and WX-554 than the HCT116 cell line (26 and 9-fold difference, respectively), which is consistent with other studies where the HT29 cell line exhibited increased sensitivity to pan class I PI3K and/or MEK inhibition, compared to the HCT116 cell line [23, 28, 29]. The observed difference could be due to the presence of a BRAF, rather than a KRAS, mutation as RAS mutations have been associated with intrinsic resistance in a previous study [30]. Alternatively, differences in cell signalling may be responsible, as sensitivity to MEK inhibition was found to correlate with strong ERK1/2 signalling and weak PI3K signalling [28]. Furthermore, the plethora of other mutations found in the HT29 and HCT116 cell lines may contribute to the difference in sensitivity [29, 31].

The combination of WX-037 and WX-554 was strongly synergistic, which is consistent with previous unpublished studies by UCB Celltech and Wilex where synergy was observed in the SK-MEL-28 melanoma, HT29 colorectal, Mia PaCa-2 pancreatic and SKOV-3 ovarian carcinoma cell lines [5]. The degree of synergy with the WX-037 and WX-554 combination was consistent with in vitro published studies using combinations of other PI3K and MEK inhibitors [2, 18, 23, 24, 32, 33].

Single agent WX-554 not only exhibited growth inhibition but also significant cytotoxicity, albeit at higher concentrations with LC_50_ values approximately 16-fold and 372-fold higher than the corresponding GI_50−_ values in the HCT116 and HT29 cell lines, respectively. This marked cytotoxicity at 10 µM is in contrast to the effect of PD 0325901 and selumetinib [23] and also to the general perspective that MEK inhibitors are cytostatic [34]. However, another study has already reported that selumetinib can cause apoptosis in some human tumour xenografts, but not in others, which was suggested to be due to differences in ERK1/2 substrate expression or differential cell signalling networks [35], and hence the result with WX-554 is not entirely unexpected.

In contrast to the effect of the MEK inhibitor, the PI3K inhibitor WX-037 at 10 µM showed only limited cytotoxicity, which is consistent with the results observed with another pan class I PI3K inhibitor pictilisib [23]. However, the marked synergy observed in the growth inhibition studies with WX-037 and WX-554 translated into cytotoxicity, albeit at a high concentration (10 µM), as there was a statistically significant increase in cytotoxicity with the combination of the Wilex PI3K and MEK inhibitors. This cytotoxicity is consistent with previous unpublished studies by UCB Celltech and Wilex where the observed growth inhibitory synergy with WX-037 and WX-554 in the SK-MEL-28 melanoma, HT29 colorectal, Mia PaCa-2 pancreatic and SKOV-3 ovarian carcinoma cell lines was found to correlate with the induction of apoptosis [5]. Similarly, published in vitro studies have shown that combined inhibition of PI3K and MEK resulted in cell death [33, 36, 37].

Cell signalling studies showed that the single agent PI3K inhibitor WX-037 caused a concentration-dependent reduction in AKT and S6 phosphorylation, which was generally enhanced by the MEK inhibitor WX-554, but that 4EBP1 phosphorylation was not inhibited, consistent with the results of published studies using pan class I PI3K, as opposed to mixed PI3K/mTOR, inhibitors [23, 32, 37, 38]. Furthermore, single agent MEK inhibitor WX-554 caused a concentration-dependent reduction in ERK phosphorylation, and the combination of WX-554 and WX-037 resulted in enhanced inhibition of ERK1/2 phosphorylation, as observed with other MEK inhibitors [23, 39, 40] and combinations [23, 33].

In the pre-clinical studies presented here, and in both pre-clinical and clinical studies reported elsewhere [5, 6], WX-554 has been shown to be non-toxic. Furthermore, single agent WX-554 induced tumour growth delay at 2 mg/kg in HCT116 colorectal tumour xenograft models, consistent with previous unpublished studies using WX-554 and published studies using other MEK inhibitors that reported tumour growth delay or stasis in vivo, with increased sensitivity in BRAF or RAS mutant cells and tumours [3–5, 25]. *In vivo* pharmacokinetic analyses revealed concentrations of WX-554 in plasma and tumour tissue increased in a dose-dependent manner and that tumour WX-554 levels generally exceeded the in vitro GI_50_ concentration, whereas plasma levels were more variable.

Single agent WX-037 was also found to be non-toxic in these studies and those reported elsewhere [5, 17], which is consistent with data for other pan class I PI3K inhibitors [41–45]. Furthermore, 50 mg/kg WX-037 generated a tumour growth delay in HCT116 colorectal tumour xenograft models, which is consistent with previous unpublished studies using WX-037 and published studies using other pan class I PI3K inhibitors that reported tumour growth delay, or in some cases tumour stasis, with greater sensitivity in PIK3CA mutant or PTEN null cells and tumours [5, 14, 15, 46–51]. Pharmacokinetic studies indicated that concentrations of WX-037 in plasma and tumour tissue increased dose dependently and that WX-037 tumour and plasma levels generally exceeded the in vitro GI_50_ concentration at an early time point (6 h), depending on the cell line and dose, whereas levels generally had decreased below the GI_50_ value by the later time point (24 h). These pharmacokinetic data are similar to those observed in HCT116 tumour xenograft-bearing mice treated with pictilisib [25].

The PK analyses indicated that there was no pharmacokinetic interaction between WX-037 and WX-554 at lower doses. However, concomitant dosing of WX-037 with the high dose (5 mg/kg) of WX-554 caused a dose-dependent decrease in early time point (6 h) WX-554 tumour and plasma concentrations, and a dose-dependent increase in late time point (24 h) WX-554 levels, suggesting that the presence of WX-037 may delay the uptake of WX-554, leading to higher WX-554 levels at 24 h. Furthermore, concomitant WX-554 dosing with high doses (100 mg/kg) of WX-037 caused a dose-dependent decrease in early time point (6 h) WX-037 tumour and plasma concentrations; however, this effect was only significant in the HCT116 tumour tissue due to variation in the data. The pharmacokinetic interaction between higher doses of WX-037 and WX-554 is in contrast to the results obtained with the combination of PD 0325901 and pictilisib in HCT116 tumour xenograft-bearing mice [25], where there was no significant pharmacokinetic interaction, and to pre-clinical and clinical reports that there was no pharmacokinetic interaction between the PI3K and MEK inhibitors, pictilisib and cobimetinib [52, 53]. However, a recent Phase Ib study using a combination of the PI3K inhibitor buparlisib and the MEK inhibitor trametinib reported a potential pharmacokinetic interaction, which was suggested to be due to the variability of the accumulation of the MEK inhibitor as no clear mechanism could be identified [54].

In HCT116 colorectal tumour xenografts, the combination of 50 mg/kg WX-037 and 2 mg/kg WX-554 was non-toxic and caused tumour stasis and enhanced tumour growth delay compared to either single agent at the same dose. This improved activity is consistent with previous studies using combinations of PI3K and MEK inhibitors in a range of pre-clinical human tumour xenograft and mouse models [19, 21, 22, 25, 55–58]. In a previous study using the MEK inhibitor, PD 0325901, and the PI3K inhibitor, GDC-0941, the cell line-dependent differences in sensitivity to the inhibitors determined in the in vitro studies did not correlate with the results observed in the in vivo studies [23, 25], suggesting that HT29 xenografts would not necessarily of been more sensitive to the inhibitors in vivo. Nevertheless, previous studies by UCB and Wilex have demonstrated that WX-554 treatment is able to induce tumour growth delay in HT29 xenografts [5].

The improved efficacy using combinations of PI3K and MEK inhibitors in pre-clinical studies is being translated into clinical trials [45, 54, 59], and there are currently at least 7 ongoing or completed Phase 1b clinical studies [9]. However, there have been issues with the tolerability of the combination treatment in two phase 1b trials combining the MEK inhibitor trametinib, with either the pan-PI3K inhibitor buparlisib, or the PI3K/mTOR inhibitor GSK2126458, where the former study showed promising anti-tumour activity but frequent dose interruptions and reductions were necessary due to toxicity [54]. The latter study was terminated due to poor tolerability and efficacy [9, 60]. These results suggest that the choice of the PI3K and MEK inhibitor to be combined will be crucial to the success of the treatment, due to subtle differences in the pharmacology of the inhibitors leading to differing toxicity profiles. Overall, these studies characterize, for the first time, the in vitro and in vivo efficacy of the PI3K inhibitor WX-037 and the MEK inhibitor WX-554, as single agents and in combination. Furthermore, these studies illustrate that dual targeting of PI3K and MEK can induce synergy in vitro which translates to marked tumour growth delay in vivo.

## Electronic supplementary material

Below is the link to the electronic supplementary material.

Supplementary material 1 (PDF 520 kb)

Supplementary material 2 (PDF 81 kb)
