# Supplementary material for: Enhanced anti-tumour activity of the combination of the novel MEK inhibitor WX-554 and the novel PI3K inhibitor WX-037
Source: Cancer Chemother Pharmacol. 2016 Nov 11;78(6):1269–81. doi: 10.1007/s00280-016-3186-4 (PMC5114336; doi:10.1007/s00280-016-3186-4)
Supplement: Supplementary file 1 — Supplementary material 1 (PDF 520 kb) [file 280_2016_3186_MOESM1_ESM.pdf]

Supplementary Figure 1

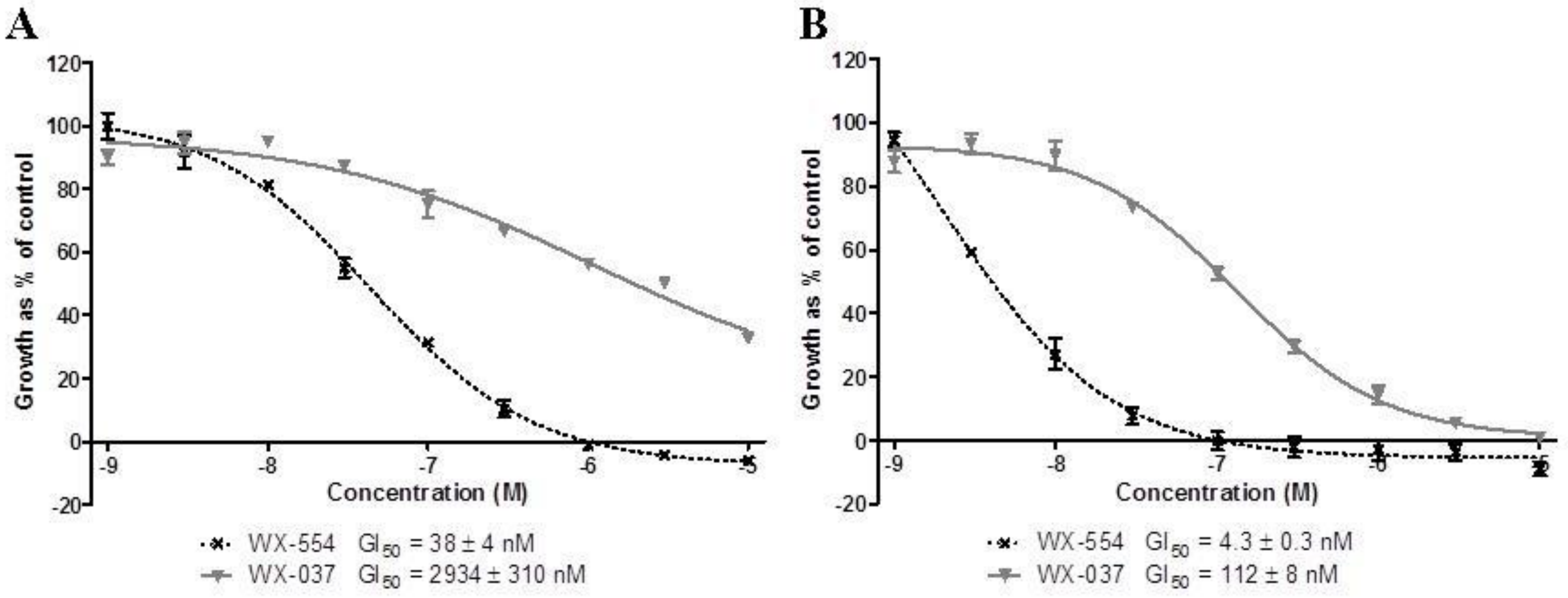

Supplementary Table 1

	Drug Concentration (Fraction of GI_{50})	Fraction affected by dose	Combination Index (CI)	Synergism/Antagonism	
HCT116	0.25	0.807	0.167	++++	Strong synergism
	0.5	0.949	0.099	+++++	Very strong synergism
	1	0.979	0.099	+++++	Very strong synergism
	2	1.008	0.014	+++++	Very strong synergism
	4	1.016	0.000	+++++	Very strong synergism
HT29	0.25	0.355	0.791	++	Moderate synergism
	0.5	0.66	0.455	+++	Synergism
	1	0.901	0.205	++++	Strong synergism
	2	1.006	0.040	+++++	Very strong synergism
	4	1.034	0.000	+++++	Very strong synergism

Supplementary Figure 2

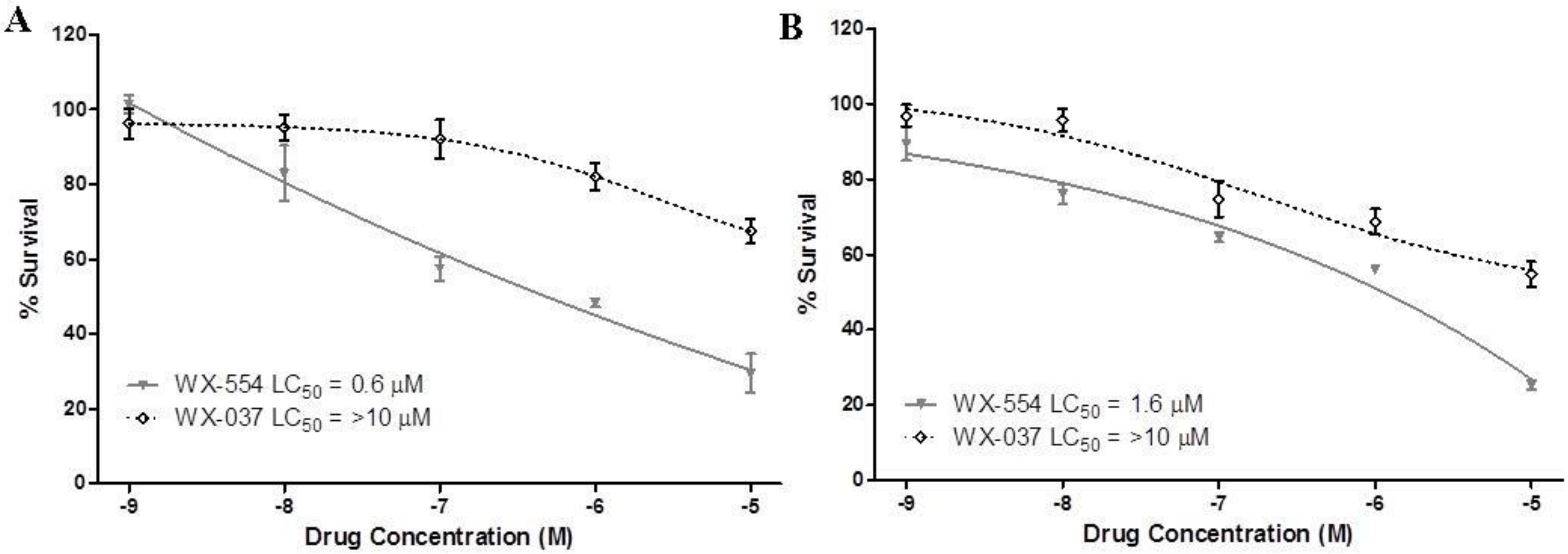

Supplementary Figure 3

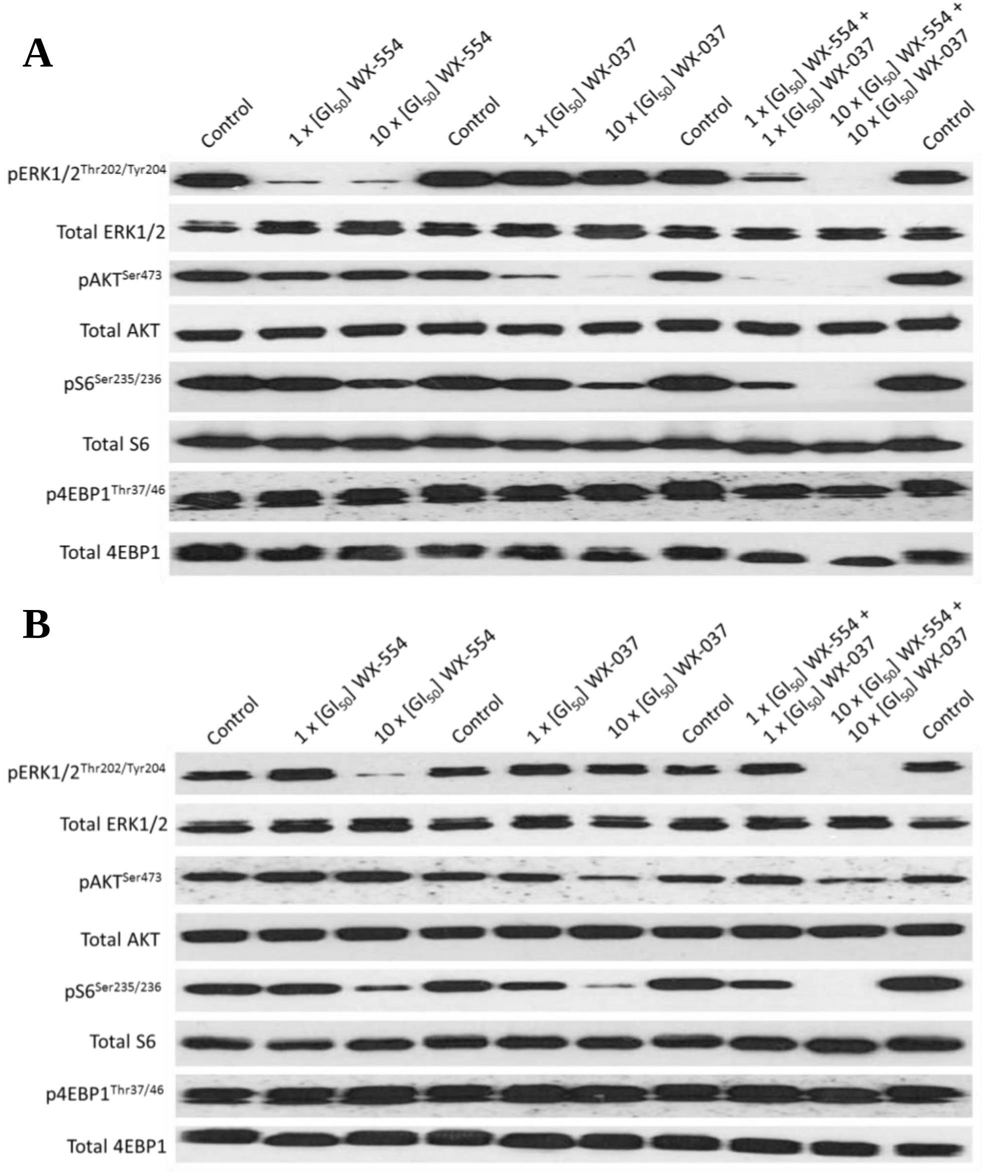

Supplementary Figure 4

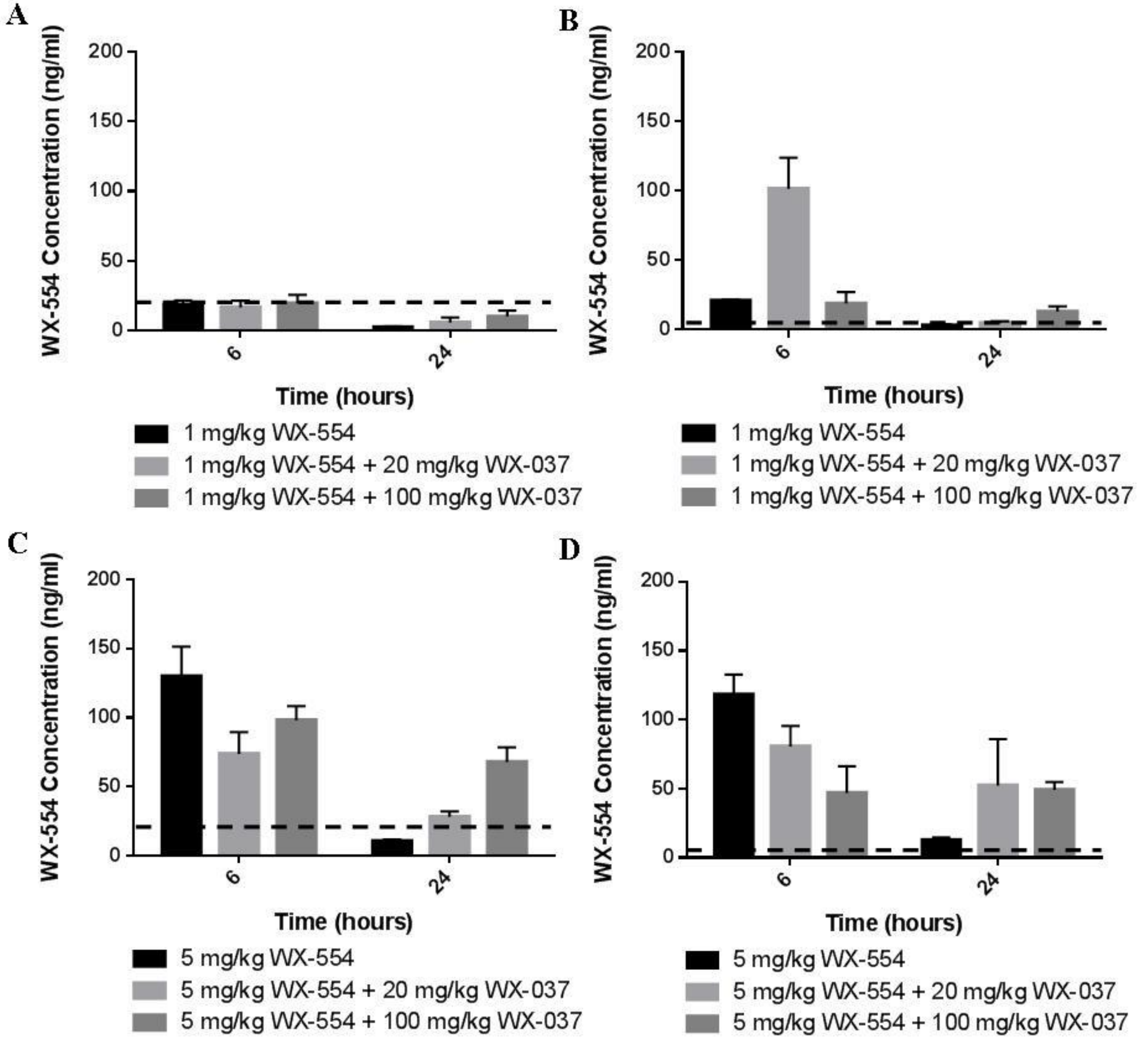

Supplementary Figure 5

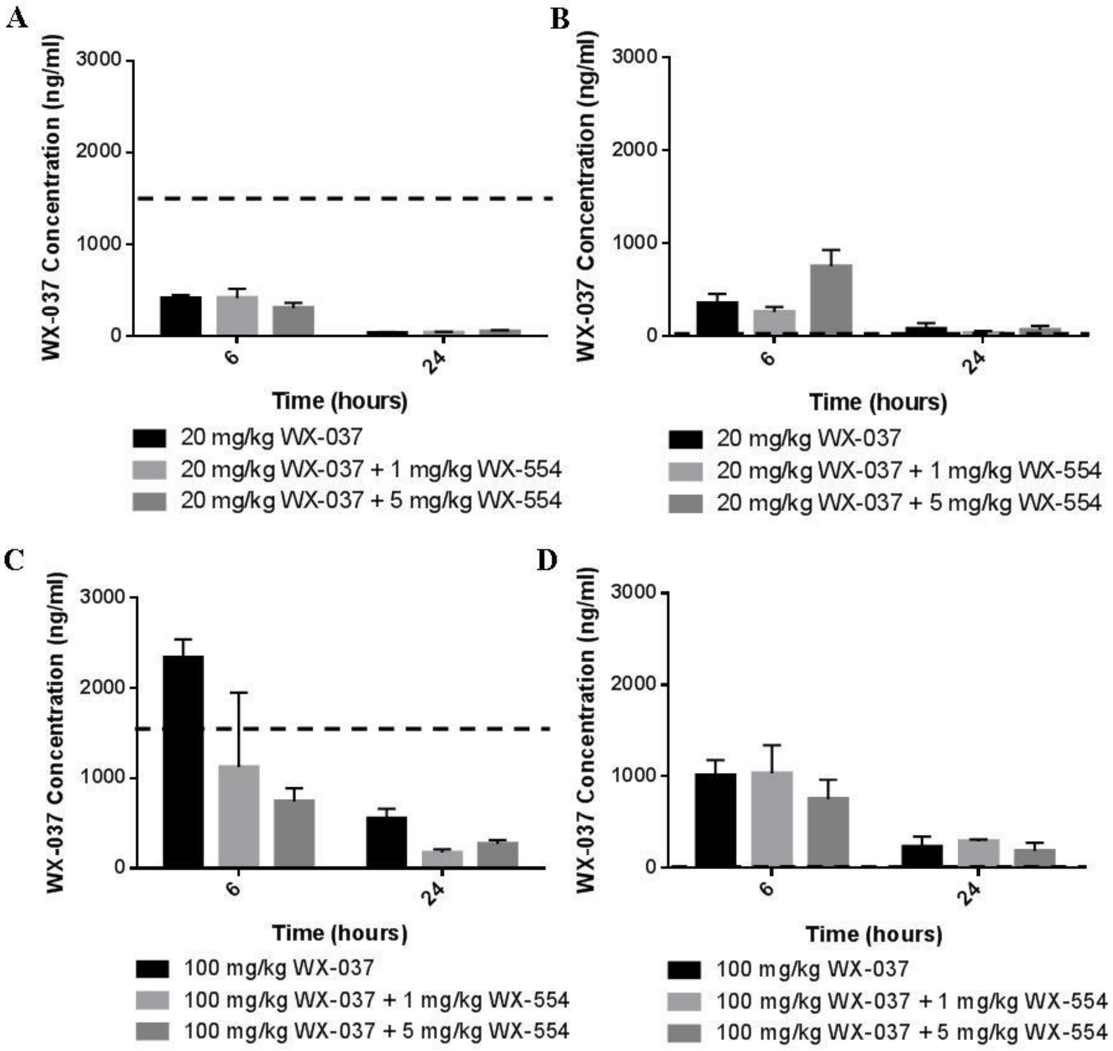

Supplementary Table 2

A

Dose		Tumour Xenograft	WX-554 Concentration ± SD (ng/ml)			
			Tumour		Plasma	
			6 hours	24 hours	6 hours	24 hours
1 mg/kg WX-554	Alone	HCT116	478 ± 97	309 ± 30	19 ± 5	2 ± 1
		HT29	510 ± 40	419 ± 134	21 ± 1	3 ± 1
	+ 20 mg/kg WX-037	HCT116	184 ± 62	141 ± 58	17 ± 9	6 ± 6
		HT29	1180 ± 597	447 ± 80	101 ± 39	5 ± 3
	+ 100 mg/kg WX-037	HCT116	319 ± 139	464 ± 263	20 ± 10	10 ± 7
		HT29	159 ± 102	407 ± 66	19 ± 14	13 ± 7
5 mg/kg WX-554	Alone	HCT116	1724 ± 442	360 ± 223	130 ± 37	11 ± 2
		HT29	2748 ± 378	1916 ± 207	118 ± 25	13 ± 3
	+ 20 mg/kg WX-037	HCT116	673 ± 453	1240 ± 282	74 ± 27	28 ± 7
		HT29	1143 ± 416	2493 ± 979	81 ± 26	52 ± 58
	+ 100 mg/kg WX-037	HCT116	804 ± 353	1583 ± 369	98 ± 18	68 ± 19
		HT29	446 ± 239	2423 ± 584	47 ± 33	49 ± 10

B

Dose		Tumour Xenograft	WX-037 Concentration ± SD (ng/ml)			
			Tumour		Plasma	
			6 hours	24 hours	6 hours	24 hours
20 mg/kg WX-037	Alone	HCT116	410 ± 64	34 ± 16	543 ± 98	1 ± 0
		HT29	354 ± 175	78 ± 104	451 ± 151	1 ± 0
	+ 1 mg/kg WX-554	HCT116	413 ± 173	36 ± 19	506 ± 238	4 ± 4
		HT29	259 ± 96	33 ± 37	322 ± 79	10 ± 12
	+ 5 mg/kg WX-554	HCT116	306 ± 96	50 ± 26	417 ± 125	4 ± 2
		HT29	752 ± 308	66 ± 78	874 ± 163	38 ± 48
100 mg/kg WX-037	Alone	HCT116	2337 ± 350	554 ± 186	2786 ± 1193	153 ± 97
		HT29	1011 ± 293	233 ± 185	1458 ± 675	283 ± 177
	+ 1 mg/kg WX-554	HCT116	1121 ± 1432	174 ± 67	1241 ± 1452	176 ± 212
		HT29	1034 ± 533	292 ± 35	1206 ± 836	483 ± 310
	+ 5 mg/kg WX-554	HCT116	740 ± 253	272 ± 73	1103 ± 390	462 ± 282
		HT29	755 ± 361	187 ± 151	706 ± 686	164 ± 58
