# Supplementary material for: Enhanced anti-tumour activity of the combination of the novel MEK inhibitor WX-554 and the novel PI3K inhibitor WX-037
Source: Cancer Chemother Pharmacol. 2016 Nov 11;78(6):1269–81. doi: 10.1007/s00280-016-3186-4 (PMC5114336; doi:10.1007/s00280-016-3186-4)
Supplement: Supplementary file 2 — Supplementary material 2 (PDF 81 kb) [file 280_2016_3186_MOESM2_ESM.pdf]

Supplementary Information

Supplementary Figure 1: Growth inhibition induced by the PI3K inhibitor WX-037 and the MEK inhibitor WX-554 in the HCT116 and HT29 cell lines. HCT116 (**A**) and HT29 (**B**) cells were treated with the indicated concentrations of the inhibitor for 72 hours, and an SRB assay was subsequently performed. Growth is presented as a percentage of the control, in which cells were treated with 0.5% (v/v) DMSO. Points represent the mean of ≥3 independent experiments ± standard error. Results were used to calculate the GI_{50} for the inhibitor and this value ± standard error is displayed below the graph. The line was fitted using non-linear regression analysis.

Supplementary Table 1: Synergy analysis of the *in vitro* interaction of the PI3K inhibitor WX-037 in combination with the MEK inhibitor WX-554 in the HCT116 and HT29 cell lines. Median effect analysis was used to evaluate the interaction between the WX-037 and WX-554 combination based on the data in Figure 1. Values represent the mean of 3 independent experiments.

Supplementary Figure 2: **Cell survival after 72 hours exposure to the PI3K inhibitor WX-037 and the MEK inhibitor WX-554 in the HCT116 and HT29 cell lines.** HCT116 (**A**) and HT29 (**B**) cells were treated with the indicated concentrations of the inhibitors for 72 hours, and cell survival was subsequently determined by clonogenic assay after 10-14 days of colony growth. Survival is presented as a percentage of the control, in which cells were treated with 0.5% (v/v) DMSO. Points represent the mean of 3 independent replicates ± standard error. Lines were fitted using non-linear regression analysis.

Supplementary Figure 3: Effect of the PI3K inhibitor WX-037 and the MEK inhibitor WX-554, as single agents and in combination, on PI3K/AKT and MAPK signal transduction in the HCT116 and HT29 cell lines. HCT116 (**A**) and HT29 (**B**) cells were treated with the indicated concentrations of the inhibitors, derived from Supplementary Figure 1, alone or in combination, for 24 hours. Cell lysates were subjected to electrophoresis, followed by Western blotting using the indicated phospho-specific antibodies. Blots were then stripped and re-probed with the corresponding total antibody to confirm equal protein loading. Data shown are representative of ≥3 independent experiments.

Supplementary Figure 4: Concentrations of the MEK inhibitor WX-554 alone and in combination with the PI3K inhibitor WX-037 in plasma from mice bearing HCT116 or HT29 human tumour xenografts. Plasma concentrations of WX-554 measured by LC-MS/MS from HCT116 (**A** and **C**) and HT29 (**B** and **D**) tumour xenograft-bearing mice at the indicated time points after a single p.o. dose of 1 mg/kg (**A** and **B**) or 5 mg/kg (**C** and **D**) WX-554 alone or combined with 20 or 100 mg/kg WX-037. Data are presented as the mean concentration from 3 mice in each group ± standard error. Horizontal dashed lines indicate the *in vitro* GI_{50} concentration for the respective cell line, calculated from Supplementary Figure 1.

Supplementary Figure 5: Concentrations of the PI3K inhibitor WX-037 alone and in combination with the MEK inhibitor WX-554 in plasma from mice bearing HCT116 or HT29 human tumour xenografts. Plasma concentrations of WX-037 measured by LC-MS/MS from HCT116 (**A** and **C**) and HT29 (**B** and **D**) tumour xenograft-bearing mice at the indicated time points after a single p.o. dose of 20 mg/kg (**A** and **B**) or 100 mg/kg (**C** and **D**) WX-037 alone or combined with 1 or 5 mg/kg WX-554. Data are presented as the mean concentration from 3 mice in each group ± standard error. Horizontal dashed lines indicate the *in vitro* GI_{50} concentration for the respective cell line, calculated from Supplementary Figure 1.

Supplementary Table 2: Plasma and tumour concentrations of the MEK inhibitor WX-554 and the PI3K inhibitor WX-037, alone and in combination, in mice bearing HCT116 or HT29 human tumour xenografts. Plasma and tumour concentrations of WX-554 and WX-037 measured by LC-MS/MS from HCT116 and HT29 tumour xenograft-bearing mice at the indicated time points after a single p.o. dose of 1 mg/kg or 5 mg/kg WX-554 alone or combined with 20 or 100 mg/kg WX-037 (**A**) or 20 mg/kg or 100 mg/kg WX-037 alone or combined with 1 or 5 mg/kg WX-554 (**B**). Data are presented as the mean concentration from 3 mice in each group ± standard deviation. Drug concentrations which are at the GI_{50} value ± 1SD for the respective cell line are shown in black; concentrations exceeding this value are shown in green, and those below in red.
